# Risk Factors Associated With Primary Care–Reported Domestic Violence for Women Involved in Family Law Care Proceedings: Data Linkage Observational Study

**DOI:** 10.2196/42375

**Published:** 2023-05-24

**Authors:** Rhodri D Johnson, Lucy J Griffiths, Laura E Cowley, Karen Broadhurst, Rowena Bailey

**Affiliations:** 1 Population Data Science Swansea University Medical School Swansea United Kingdom; 2 Centre for Child & Family Justice Research Sociology Bowland College, Lancaster University Lancaster United Kingdom

**Keywords:** data linkage, domestic violence, domestic abuse, health data, family justice data

## Abstract

**Background:**

Domestic violence and abuse (DVA) has a detrimental impact on the health and well-being of children and families but is commonly underreported, with an estimated prevalence of 5.5% in England and Wales in 2020. DVA is more common in groups considered vulnerable, including those involved in public law family court proceedings; however, there is a lack of evidence regarding risk factors for DVA among those involved in the family justice system.

**Objective:**

This study examines risk factors for DVA within a cohort of mothers involved in public law family court proceedings in Wales and a matched general population comparison group.

**Methods:**

We linked family justice data from the Children and Family Court Advisory and Support Service (Cafcass Cymru [Wales]) to demographic and electronic health records within the Secure Anonymised Information Linkage (SAIL) Databank. We constructed 2 study cohorts: mothers involved in public law family court proceedings (2011-2019) and a general population group of mothers not involved in public law family court proceedings, matched on key demographics (age and deprivation). We used published clinical codes to identify mothers with exposure to DVA documented in their primary care records and who therefore reported DVA to their general practitioner. Multiple logistic regression analyses were used to examine risk factors for primary care–recorded DVA.

**Results:**

Mothers involved in public law family court proceedings were 8 times more likely to have had exposure to DVA documented in their primary care records than the general population group (adjusted odds ratio [AOR] 8.0, 95% CI 6.6-9.7). Within the cohort of mothers involved in public law family court proceedings, risk factors for DVA with the greatest effect sizes included living in sparsely populated areas (AOR 3.9, 95% CI 2.8-5.5), assault-related emergency department attendances (AOR 2.2, 95% CI 1.5-3.1), and mental health conditions (AOR 1.7, 95% CI 1.3-2.2). An 8-fold increased risk of DVA emphasizes increased vulnerabilities for individuals involved in public law family court proceedings.

**Conclusions:**

Previously reported DVA risk factors do not necessarily apply to this group of women. The additional risk factors identified in this study could be considered for inclusion in national guidelines. The evidence that living in sparsely populated areas and assault-related emergency department attendances are associated with increased risk of DVA could be used to inform policy and practice interventions targeting prevention as well as tailored support services for those with exposure to DVA. However, further work should also explore other sources of DVA, such as that recorded in secondary health care, family, and criminal justice records, to understand the true scale of the problem.

## Introduction

### Background

Domestic violence and abuse (DVA) is known to have long-term and wide-ranging detrimental impacts on the health and well-being of children and families [1-7]. The UK Domestic Abuse Act 2021 [8] defines “abusive behaviour” as occurring between “personally connected” parties aged ≥16 years and includes “physical or sexual abuse; violent or threatening behaviour; controlling or coercive behaviour; economic abuse; and psychological, emotional, or other abuse.”

Annual DVA statistics are published for Wales and England by the Office for National Statistics (ONS), using information from the Crime Survey for England and Wales (CSEW). The latest available DVA data from the CSEW highlighted that women are much more likely to be exposed to DVA than men, with an estimated 7.3% of women (1.6 million) and 3.6% of men (757,000) aged 16 to 74 years experiencing DVA in the year ending March 2020 [9]. Research studies report varying estimates of the prevalence of DVA toward women in the United Kingdom; in 1 systematic review, the lifetime prevalence of partner violence against women in the general UK population ranged from 13% to 31%, whereas the 1-year prevalence ranged from 4.2% to 6% [10]. The 1-year prevalence of DVA for women attending a general practitioner (GP) in the United Kingdom was estimated to be 17% in a cross-sectional survey study [11] and 0.15% in a recent data linkage study that used Read (Read refers to originator Dr James Read) codes (hierarchical nomenclature used to record clinical information) to search for records of DVA within GP administrative data’ [12]. Another recent primary care data linkage study estimated that the prevalence of women in the United Kingdom aged ≥18 years recorded as having lifetime experience of DVA in 2017 was 0.37% [13].

Hard-to-reach populations are often excluded from DVA research and statistics [14,15]; for example, the CSEW excludes those living in nonpermanent households and therefore risks excluding individuals considered vulnerable in women’s refuges as well as homeless populations [14]. One such group that is considered vulnerable and often hard to reach comprises individuals involved in public law family court proceedings. When a child is identified as having experienced, or is at risk of experiencing, significant harm at the hands of a parent or caregiver, the local authority may issue care or supervision proceedings under section 31 of the Children Act (1989; s.31). Since the advent of the Adoption and Children Act 2002, harm to children now includes being exposed to, or witnessing, DVA [16]. This change to the definition of significant harm reflected growing awareness of the negative impact of DVA on children and families. Nevertheless, this growing recognition has not been matched by sufficient analysis of DVA for families involved with the family courts.

There is an important evidence base on the heightened health vulnerabilities of parents and children involved in public family law proceedings, including high rates of mental health need and substance use [17-25]. However, published research on DVA using large-scale data is more limited, despite DVA being classed as a major public health concern [26]. Drawing on the available research, 2 studies [27,28] conducted in England suggest that the prevalence of DVA in women involved in s.31 care proceedings is much higher than that in women in the general population. In a study of a sample of 386 s.31 applications made by 15 English local authorities, a little more than half of the mothers (51.1%) had their concerns about DVA recorded by children’s services [27]. Similarly, a case file analysis of a random sample of 354 mothers involved in recurrent care proceedings in England from 50 local authorities revealed that 65% of the mothers had been exposed to DVA by the time of the initial proceedings [28]. Local authorities have highlighted that DVA is one of the most important drivers of the increased demand for children’s social care services in recent years [29]. Annual statistics on children “in need” assessments in England have consistently demonstrated that DVA within the household is the most common concern identified at the end of assessments, and it remained the most common factor in 2020-2021 [30,31]. Although comparable data from Wales are unavailable, a survey of adverse childhood experiences conducted in 2015 reported that 16% of the Welsh adult population were exposed to DVA during childhood [32].

Regarding studies using population-level data to understand DVA in the broader child welfare population, a small number of studies are notable. A recent data linkage study reported that children of mothers with experience of DVA in Manitoba, Canada, were significantly more likely to be receiving services from child welfare organizations than children of mothers who reported no prior experience of DVA [15]. Another longitudinal ecological study found that increases in the rate of infant entry into care over time in England were associated with an increased prevalence of maternal history of adversity-related hospital admissions, including hospital admissions related to exposure to DVA [33]. A further study examined patterns of co-occurrence and severity of DVA, substance misuse, and depression in female caregivers involved with child protective services in the United States; one-third of the sample reported high rates of all factors, including both minor and severe DVA, whereas only 9% reported none [34]. Among women with experience of DVA, mothers whose children were taken into care had more children, were more likely to have self-reported addictions, and had significantly less education than mothers without child protective services involvement. In addition, those with exposure to DVA and with children in care reported significantly more physical abuse from their partners, more psychological distress, and lower quality of life than mothers whose children were not in care; however, there were no statistically significant associations with clinical depression or posttraumatic stress disorder [35].

Risk factors for DVA among women in the general population are listed in the National Institute for Health and Care Excellence (NICE) public health guidance on identifying, preventing, and reducing DVA [36], and they include age (women aged between 16 and 24 years are at higher risk than older women), long-term illness or disability, poor mental health, being separated from a partner, pregnancy, postnatal depression, and substance misuse. Other risk factors reported in the literature include intentional and nonintentional injury hospitalizations [15], unemployment, and living in a low-income or single-parent household [37].

### Objectives

The availability of routinely collected population-level administrative data for research provides the opportunity to study cohorts of populations that are hard to reach or considered high risk [38]. This study analyzed administrative records held within the Secure Anonymised Information Linkage (SAIL) Databank [39-41] for a cohort of mothers involved in public family law court proceedings and a matched group of mothers in the general population of Wales. The primary aim was to investigate risk factors associated with primary care–recorded DVA for women involved in public family law proceedings. In addition, we aimed to (1) estimate involvement in public family law proceedings as a risk factor for primary care–recorded DVA compared with a comparison group, (2) estimate the population and cohort prevalence of primary care–recorded DVA, and (3) evaluate whether routinely collected data could be used to create and identify additional risk factors associated with DVA.

## Methods

### Study Design and Data Sources

#### Overview

This is a population-scale, individual-level cohort study that examines risk factors of primary care–recorded DVA for mothers in Wales. The study analyzed data held within the SAIL Databank [39-41], which contains extensive deidentified health and administrative data for the population of Wales [42]. Anonymized linkage fields (ALFs) are assigned to successfully matched individuals, which permits data linkage across multiple data sets. Individuals can also be linked to registered addresses via a residential ALF (RALF) [43,44]. For this study, we used data from multiple linked data sets, described in the following subsections. The SAIL Databank was interrogated using Db2 SQL.

#### Children and Family Court Advisory and Support Service Data Set

The Children and Family Court Advisory and Support Service (Cafcass Cymru) is an organization appointed by the family court to ensure that decisions are made in the best interests of the child [45]. This data set has been described elsewhere [46] and contains information on individuals involved in public law family court proceedings, including court application dates, maternal age, age of the youngest child, and number of children.

#### Welsh Demographic Service Dataset

The Welsh Demographic Service Dataset (WDSD) contains demographic and address records for all individuals resident in Wales and registered with a GP.

#### Welsh Index of Multiple Deprivation 2014

The Welsh Index of Multiple Deprivation (WIMD) 2014 contains deprivation quintiles corresponding to all lower layer super output areas (LSOAs; geographic units comprising approximately 1600 individuals) in Wales [47].

#### Rural Urban Classification Data Set

The Rural-Urban Classification data set contains information on rural-urban classifications corresponding to all LSOAs in Wales [48].

#### Welsh Longitudinal General Practice Data Set

The Welsh Longitudinal General Practice data set contains attendance and clinical information for all GP interactions in Wales, including symptoms, diagnoses, and prescriptions [49]. GPs opt in to providing data to the SAIL Databank; currently, the SAIL Databank contains primary care data for approximately 80% of the Welsh population, and the available data are representative of the entire population of Wales with respect to age, sex, and deprivation [50].

#### Patient Episode Database for Wales

The Patient Episode Database for Wales (PEDW) contains attendance and clinical information for all hospital admissions in Wales, including data regarding diagnoses [51].

#### Emergency Department Data set

The Emergency Department Dataset (EDDS) contains administrative and clinical information for all emergency department (ED) attendances in Wales [52].

### Study Populations

#### Mothers Involved in Public Law Family Court Proceedings (Cohort)

**Figure 1:**
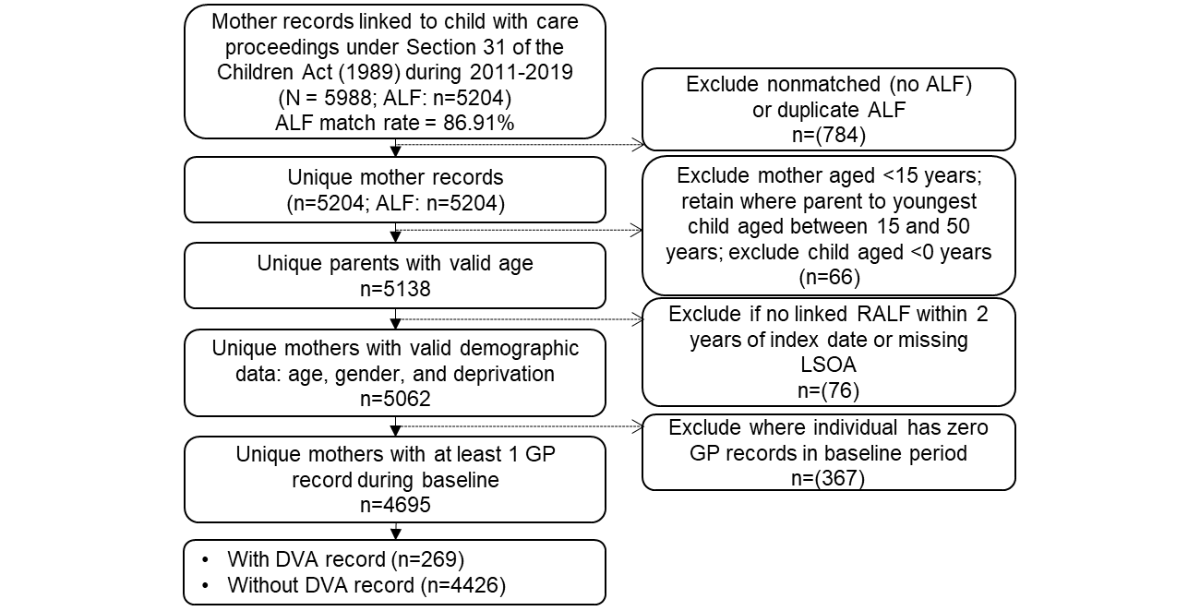
Study cohort selection flow diagram. ALF: anonymized linkage field; DVA: domestic violence and abuse; GP: general practitioner; LSOA: lower layer super output area; RALF: residential anonymized linkage field.

The methods used to create the study cohort and population comparison group were based on those used in an earlier study examining health vulnerabilities of parents involved in care proceedings in Wales [19,25]. For that study, the Cafcass Cymru data were used to select parents (both mothers and fathers) of children involved in s.31 care proceedings in Wales between 2011 and 2019, including only those parents with an ALF and valid demographic data. For this study, the sample was restricted to mothers only. The final cohort of mothers involved in public law family court proceedings included only those who had GP data available during the 2-year baseline period before their first s.31 proceeding court application date (index date) between 2011 and 2019 (Figure 1).

#### General Population Comparison Group

For the previous study [19,25], a general population comparison group of parents (both mothers and fathers) who had not been involved in care proceedings was derived. As there are no specific *parent identifiers* within the SAIL Databank, this study selected individuals from the WDSD and then used a published algorithm [44] to identify family households in Wales, retaining records relating to parents of children aged <18 years at a fixed index date (the study midpoint: July 1, 2015). Only parents with valid demographic data were retained. Parents were then matched to the cohort of parents involved in care proceedings on key demographic variables (age and area-level deprivation). As with the cohort selection, for this study, this sample was restricted to mothers who had GP data available during the baseline period.

### Measures

Hospital data for inpatient and day case activity were extracted from the PEDW; data from any episode and any diagnostic position were included. ED data were extracted from the EDDS for new attendances only. All measures were calculated across a 2-year baseline period before the index date.

### Demographics

Mothers were linked at the individual level to health and demographic data sets using the ALF, and RALFs were linked to LSOAs to obtain area-level measures. Age, number of children, and youngest child’s age were calculated as of the index date and derived from the Cafcass Cymru and WDSD data for the cohort and comparison mothers, respectively. Deprivation quintiles (most to least deprived using the WIMD income domain) and ONS urban/rural classifications (further divided into sparsely populated areas and nonsparsely populated areas) were used for area-level measures.

### DVA Measure

The DVA measure was represented as a binary variable where 1 indicated that DVA was recorded, based on the presence of any codes indicative of DVA in a GP-validated clinical Read code list [12]. The Read code list is provided in Multimedia Appendix 1 [12].

### Risk Factors

Risk factors were created and aimed to represent NICE risk factors. Age was categorized as <25 years or ≥25 years*.* Binary variables were derived for health measures, where 1 indicated the presence of at least 1 record of interest during baseline measurement. An intellectual disability variable was created from Welsh Longitudinal General Practice data using a validated Read code list [53] as a proxy measure for long-term illness. Mental ill-health (including depression, anxiety, attention-deficit/hyperactivity disorder [ADHD], and autism) and substance use variables (suggesting problematic use of illicit drugs and alcohol) were created using a validated code list [54] for any hospital admissions (PEDW) or GP records. A *pregnant or recent childbirth* measure was created and recorded for mothers with at least 1 hospital admission record during baseline categorized under the International Classification of Diseases, 10th Revision (ICD-10), as “Pregnancy, childbirth and the puerperium” [55]. A proxy measure of *house moves* was created as a binary variable to represent the *separation* risk factor [56,57] using the WDSD, where 1 represents any mother with >1 address registration during the baseline period.

Additional risk factors not included in NICE guidelines were added: *assault-related emergency department attendances* using the EDDS attendance category, *more than one child* and *child aged <5 years 5* using Cafcass Cymru and WDSD data, *living in the 2 most deprived quintiles* using the WIMD, and ONS rurality measures further classified into sparsely or nonsparsely populated areas.

### Statistical Analysis

The initial analyses, referred to as *population-risk analyses*, included a general population group of mothers categorized into 2 groups: mothers *with* family court involvement and those *without* family court involvement. A second set of analyses were restricted to family court mothers, grouped by the presence or absence of primary care–recorded DVA, referred to as *cohort-risk analyses*. We examined demographic differences between the groups using the Pearson chi-square test for categorical variables and the Welch 2-tailed *t* test for numeric variables and reported the corresponding effect sizes and *P* values.

We calculated the prevalence of primary care–recorded DVA for the cohort and the general population comparison group within a 2-year period before the index date. Univariable and multivariable logistic regression analyses were used to calculate differences in primary care–recorded DVA between the groups and are presented as unadjusted odds ratios (ORs) and adjusted ORs (AORs) with 95% CIs to test for associations between primary care–recorded DVA and risk factors. All risk factors that were created to represent those from the NICE guidelines were included with no further variable selection in model 1 and model 2 for the population-risk analyses and the cohort-risk analyses, respectively. This was to allow for comparison of results between the population-risk analyses and the cohort-risk analyses. A final adjusted model, model 3, was created with the aim of finding the best-fitting model for the cohort mothers balanced with achieving a parsimonious model, combining NICE risk factors and additional risk factors. Model 3 variable entry was based on the inclusion of model 2 terms with *P* values <.20, along with inclusion of additional risk factors, which improved the model fit using the area under the curve (AUC).

### Ethics Approval and Compliance Rules

The SAIL Databank uses an independent governance review panel (IGRP) that assesses each application for approval. For general data processing activities, the SAIL Databank relies on General Data Protection Regulation (GDPR) Articles 6 and 9 (provisions for a task carried out in the public interest). Cafcass lawfully shares information about family court proceedings for approved research within the SAIL Databank under Practice Direction 12G–Communication of Information (paragraph 2.1). 

## Results

### Demographic Characteristics

After cohort creation, the total sample of 237,866 consisted of 4695 (1.97%) cohort mothers (ALF match rate: 5204/5988, 86.91%; Figure 1) and 233,171 (98.03%) comparison mothers. The 2-year prevalence of DVA was 5.73% (269/4695) for the cohort and 0.27% (626/233,171) for the general population comparison group.

The demographic characteristics varied considerably between the cohort mothers and the general comparison group with statistically significant differences in age, deprivation, and rurality measures (Table 1). Cohort mothers were approximately 9 years younger, more likely to live in deprived areas (2355/4695, 50.16%, vs 53,849/233,171, 23.09%, in the most deprived quintile), and more likely to live in *urban city and town* areas than more rural and sparsely populated areas. No within-cohort differences were observed for deprivation, but there were statistically significant differences in terms of rurality measures, with mothers with primary care–recorded DVA more likely to live in sparsely populated areas than those with no primary care record of DVA (50/269, 18.6%, vs 282/4426, 6.37%, respectively). Mothers exposed to DVA were also on average slightly younger (27.8, SD 7.0, years vs 29.0, SD 8.2, years), which was statistically significant but with a small effect size (t_314_=2.7).

**Table 1:** Number (and percentage) of study groups by demographic characteristics and risk factors included in the population-risk analyses and the cohort-risk analyses (N=237,866).

Variable			General comparison group (n=233,171)		Cohort (total; n=4695)		Cohort: with DVA^a^ (n=269)		Cohort: no DVA (n=4426)
DVA outcome, n (%)			626 (0.3)		269 (5.7)		269 (100)		N/A^b^
Aged <25 years, n (%)			11,230 (4.8)		1607 (34.2)		94 (34.9)		1513 (34.2)
Intellectual disability, n (%)			197 (0.1)		120 (2.6)		11 (4.1)		109 (2.5)
Mental health conditions, n (%)			39,539 (17)		2638 (56.2)		187 (69.5)		2451 (55.4)
House moves, n (%)			48,579 (20.8)		2648 (56.4)		168 (62.5)		2480 (56)
Recent pregnancy or childbirth, n (%)			44,720 (19.2)		2739 (58.3)		168 (62.5)		2571 (58.1)
Substance use conditions, n (%)			2430 (1)		1007 (21.4)		71 (26.4)		936 (21.1)
Lives in sparsely populated area, n (%)			N/A		332 (7.1)		50 (18.6)		282 (6.4)
Assault-related ED^c^ attendance, n (%)			N/A		422 (9)		42 (15.6)		380 (8.6)
>1 child, n (%)			N/A		1849 (39.4)		135 (50.2)		1714 (38.7)
Lives in 2 most deprived quintiles, n (%)			N/A		3526 (75.1)		195 (72.5)		3331 (75.3)
Child aged <5 years, n (%)			N/A		3480 (74.1)		217 (80.7)		3263 (73.7)
Age (years), mean (SD)			38.3 (8.4)		28.9 (8.1)		27.8 (7.0)		29.0 (8.2)
**Deprivation,** **n (%)**									
	1 (most deprived quintile)	53,849 (23.1)		2355 (50.2)		131 (48.7)		2224 (50.2)	
	2	48,522 (20.8)		1171 (24.9)		64 (23.8)		1107 (25)	
	3	46,125 (19.8)		633 (13.5)		45 (16.7)		588 (13.3)	
	4	42,144 (18.1)		357 (7.6)		20 (7.4)		337 (7.6)	
	5 (least deprived quintile)	42,531 (18.2)		179 (3.8)		9 (3.3)		170 (3.8)	
**Rurality, n (%)**									
	Urban city and town	164,315 (70.5)		3703 (78.9)		175 (65.1)		3528 (79.7)	
	Urban city and town (sparse)	4055 (1.7)		126 (2.7)		23 (8.6)		103 (2.3)	
	Rural town and fringe	32,970 (14.1)		557 (11.9)		38 (14.1)		519 (11.7)	
	Rural town and fringe (sparse)	6596 (2.8)		99 (2.1)		13 (4.8)		86 (1.9)	
	Rural village and dispersed	12,167 (5.2)		103 (2.2)		6 (2.2)		97 (2.2)	
	Rural village and dispersed (sparse)	13,068 (5.6)		107 (2.3)		14 (5.2)		93 (2.1)	

^a^DVA: domestic violence and abuse.

^b^N/A: not applicable.

^c^ED: emergency department.

### Population-Risk Analyses

Compared with the general population group, the cohort had a higher proportion of mothers aged <25 years (1607/4695, 34.22%, vs 11,230/233,171, 4.82%), mothers with mental health conditions (2638/4695, 56.19%, vs 39,539/233,171, 16.96%), mothers with an intellectual disability (120/4695, 2.56%, vs 197/233,171, 0.08%), mothers with substance use conditions (1007/4695, 21.45%, vs 2430/233,171, 1.04%), mothers who had a recent pregnancy or childbirth (2739/4695, 58.34%, vs 44,720/233,171, 19.18%), and mothers who had multiple house moves (2648/4695, 56.4%, vs 48,579/233,171, 20.83%).

The unadjusted odds of mothers in the cohort having a record of DVA was almost 23 times higher than those of mothers in the general population (OR 22.8, 95% CI 19.5-26.1). All NICE risk factors were found to have statistically significant positive associations with primary care–recorded DVA with large effect sizes (Table 2). Mothers with an intellectual disability (OR 10.6, 95% CI 5.6-18.0) or substance use conditions (OR 10.3, 95% CI 8.4-12.5) were 10 times more likely to have primary care–recorded DVA than those without an intellectual disability or without a substance use condition, respectively.

Adjusting for the differences in risk factors between the groups, the cohort were 8 times more likely to have primary care–recorded DVA (AOR 8.0, 95% CI 6.6-9.7; model 1). All 6 NICE guideline risk factors retained the same direction of association with primary care–recorded DVA, albeit with smaller effect sizes, and 4 (%) remained statistically significant after adjustment: being aged <25 years, having a mental health condition, having multiple house moves, and having substance use conditions. Model fit metrics showed AUC=79% and Hosmer *χ*^2^_8_=18.4 (*P*=.02).

**Table 2:** Unadjusted and adjusted odds ratios (and 95% CIs) of self-reported primary care–recorded domestic violence and abuse (combined cohort and comparison group). Model 1: National Institute for Health and Care Excellence (NICE) guideline risk factors.

Term	Unadjusted odds ratio (95% CI)	*P* value	Model 1 adjusted odds ratio (95% CI)	*P* value
Family court involvement	22.58 (19.48-26.09)	<.001	8.00 (6.62-9.65)	<.001
Aged <25 years	4.81 (4.09-5.64)	<.001	1.48 (1.22-1.80)	<.001
Intellectual disability	10.55 (5.58-18.00)	<.001	1.55 (0.80-2.75)	.16
Mental health conditions	5.15 (4.52-5.88)	<.001	2.94 (2.55-3.40)	<.001
House moves	3.83 (3.36-4.36)	<.001	2.15 (1.86-2.50)	<.001
Recent pregnancy or childbirth	2.47 (2.15-2.82)	<.001	1.03 (0.87-1.21)	.75
Substance use conditions	10.26 (8.37-12.47)	<.001	1.90 (1.51-2.39)	<.001
Intercept	N/A^a^	N/A	0.00 (0.00-0.00)	<.001

^a^N/A: not applicable.

### Cohort-Risk Analyses

Within the cohort, comparing differences between mothers with a record of DVA and those without a record of DVA, there was a higher proportion of mothers with DVA with all risk factors, with 1 exception: there was a slightly higher proportion of mothers with no recorded DVA living in the 2 most deprived quintiles (Table 3). Unadjusted ORs indicate significantly higher risk of mental health conditions, substance use conditions, and house moves among mothers with primary care–recorded DVA than among those without primary care–recorded DVA, all with relatively small effect sizes with OR values of <2. Additional risk factors that were significantly associated with primary care–recorded DVA within the cohort included living in a sparsely populated area, assault-related ED attendances, and child-related variables (>1 child and child aged <5 years), with OR values between 1.5 and 3.4. Age indicated a protective effect (OR 0.98, 95% CI 0.97-1.0). Adjusting for all NICE risk factors in the model (model 2), only mental health remained as a significantly associated risk factor: mothers involved in the family courts with primary care–recorded DVA had 75% higher odds of having mental health issues than those without primary care–recorded DVA. House moves and substance use became nonsignificant when adjusting for all other factors. Model 2 fit metrics showed AUC=60% and Hosmer *χ*^2^_7_=5.6 (*P*=.69).

The final model 3 included 3 NICE risk factors (mental health, substance use, and house moves), along with 3 additional variables (living in a sparsely populated area, assault-related ED attendances, and having >1 child). The risk factors associated with the largest effect sizes were *living in a sparsely populated area* (AOR 3.9, 95% CI 2.8-5.5) and at least 1 assault-related ED attendance (AOR 2.2, 95% CI 1.5-3.1) for family court mothers with primary care–recorded DVA compared with mothers without primary care–recorded DVA. Mothers with primary care–recorded DVA were also 82% more likely to have >1 child (AOR 1.82, 95% CI 1.41-2.35), 69% more likely to have a mental health issue (AOR 1.69, 95% CI 1.29-2.23), and 35% more likely to have multiple house moves (AOR 1.35, 95% CI 1.04-1.8). Model 3 metrics showed AUC=66% and Hosmer *χ*^2^_7_=4.6 (*P*=.80).

**Figure 2:**
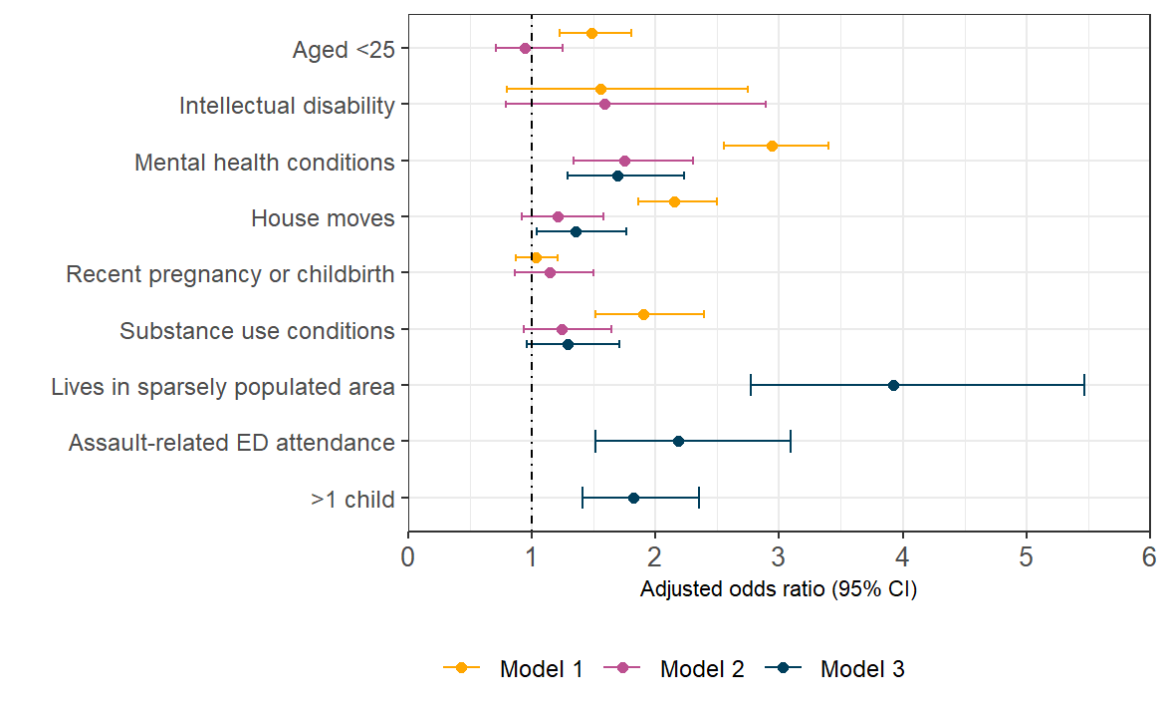
Adjusted odds ratios and 95% CIs of risk factors for domestic violence and abuse for all 3 models. ED: emergency department.

**Figure 3:**
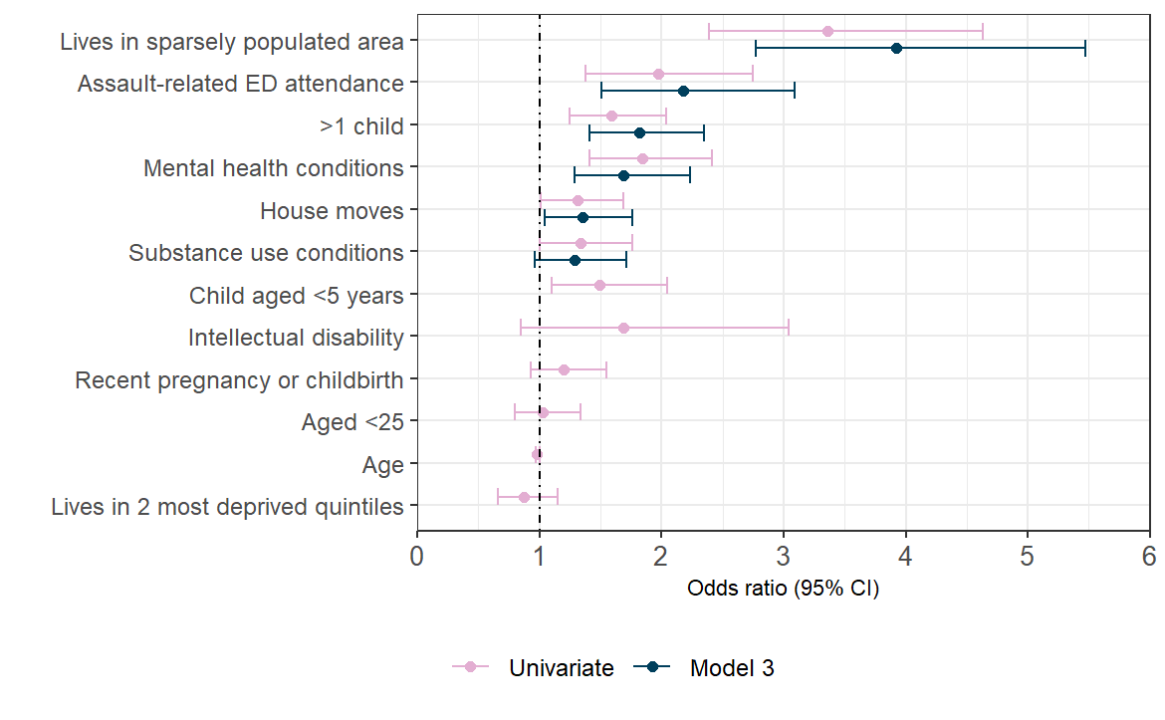
Unadjusted and adjusted odds ratios and 95% CIs for all risk factors tested within the final model 3. ED: emergency department.

Figure 2 illustrates AORs and 95% CIs of risk factors for all 3 models. The large effect size (AOR 8.00, 95% CI 6.62-9.65) for family court involvement in model 1 is excluded from Figure 2 to aid visualization of smaller effect sizes. Figure 3 shows unadjusted ORs and AORs and 95% CIs for all risk factors tested within the final model 3.

**Table 3:** Unadjusted and adjusted odds ratios (and 95% CIs) of self-reported primary care–recorded domestic violence and abuse (within-cohort comparisons). Model 2: National Institute for Health and Care Excellence (NICE) guideline risk factors only. Model 3: NICE guideline risk factors plus additional risk factors.

Term	Unadjusted odds ratio (95% CI)	*P* value	Model 2^a^ adjusted odds ratio (95% CI)	*P* value	Model 3^b^ adjusted odds ratio (95% CI)	*P* value
Aged <25 years	1.03 (0.80-1.34)	.80	0.94 (0.71-1.25)	.70	N/A^c^	N/A
Intellectual disability	1.69 (0.85-3.04)	.11	1.59 (0.79-2.89)	.15	N/A	N/A
Mental health conditions	1.84 (1.41-2.41)	<.001	1.75 (1.34-2.30)	<.001	1.69 (1.29-2.23)	<.001
House moves	1.31 (1.01-1.69)	.04	1.21 (0.92-1.58)	.17	1.35 (1.04-1.76)	.03
Recent pregnancy	1.20 (0.93-1.55)	.16	1.14 (0.86-1.50)	.37	N/A	N/A
Substance use conditions	1.34 (1.00-1.76)	.04	1.24 (0.93-1.64)	.14	1.29 (0.96-1.71)	.09
Lives in sparsely populated area	3.36 (2.39-4.63)	<.001	N/A	N/A	3.92 (2.77-5.47)	<.001
Assault-related ED^d^ attendance	1.97 (1.38-2.75)	<.001	N/A	N/A	2.18 (1.51-3.09)	<.001
>1 child	1.59 (1.25-2.04)	<.001	N/A	N/A	1.82 (1.41-2.35)	<.001
Lives in 2 most deprived quintiles	0.87 (0.66-1.15)	.31	N/A	N/A	N/A	N/A
Child aged <5 years	1.49 (1.10-2.05)	.01	N/A	N/A	N/A	N/A
Age (years), mean (SD)	0.98 (0.97-1.00)	.02	N/A	N/A	N/A	N/A
Intercept	N/A	N/A	0.03 (0.02-0.05)	<.001	0.02 (0.01-0.03)	<.001

^a^Analysis restricted to National Institute for Health and Care Excellence (NICE) guideline risk factors.

^b^Final model: National Institute for Health and Care Excellence (NICE) guideline risk factors and additional risk factors.

^c^N/A: not applicable.

^d^ED: emergency department.

## Discussion

### Principal Findings

This study found a 2-year primary care–recorded DVA prevalence of 5.73% (269/4695) and 0.27% (626/233,171) for a cohort of public law family court mothers and mothers from the general population of Wales, respectively. We found that mothers involved in the public law family courts were at increased risk of DVA, being 8 times more likely to have primary care–recorded DVA than the general population group. Factors that were significantly associated with primary care–recorded DVA in the general population group included being aged <25 years, having a record of a mental health or substance use condition, and having multiple house moves. Of these factors, only mental health remained significantly associated with primary care–recorded DVA within the cohort of mothers involved in the public law family courts. Additional variables, informed by the literature and derived from linked administrative data, were also significantly associated with primary care–recorded DVA within the cohort of family court mothers; these included living in a sparsely populated area, having an assault-related ED attendance, and having >1 child. However, deprivation, so often associated with risk of poor health, was not found to have a significant association with primary care–recorded DVA.

### Comparison With Prior Work

Our reported 2-year prevalence of primary care–recorded DVA in the general population (626/233,171, 0.27%) is in line with a 1-year prevalence of 0.1% reported in a recent study [12]. For the family court cohort, the prevalence of 5.73% (269/4695) is comparable with a previous data linkage study reporting a 1-year prevalence of DVA of 5% in mothers whose children had entered public care [58]. Furthermore, our finding that these mothers are 8 times more likely to have a record of primary care–recorded DVA than the general population group is similar to that of previous research reporting AOR values of 8.8 for an association between *child welfare involvement* and DVA screened for at childbirth [15]. Finally, our findings are similar to recent evidence showing that 4% of the women involved in *private* family law cases in Wales had exposure to DVA noted in their GP records in the year before the proceedings [59].

Our findings are consistent with those of previous studies that have highlighted the co-occurrence of DVA with mental health and substance use problems in the general population [15,60,61]. Studies have also highlighted the high prevalence and co-occurrence of DVA, poor mental health, and substance use among parents whose children have been taken into care [33,34,59,62]. However, in this study, only mental health remained significantly associated with DVA within the cohort of mothers involved with the family courts, once all other risk factors were adjusted for, and substance use was not significantly associated with DVA. Although the relationship between poor mental health and DVA is likely bidirectional, this finding emphasizes that mothers involved with the public law family courts who have experienced DVA are likely to have an additional need for mental health services and ongoing mental health support.

Maternal age is commonly reported as having negative associations with DVA, with younger age relating to increased risk [36,37]. In this study, being aged <25 years was associated with DVA in the general population group but not in the cohort of mothers involved in public law family court proceedings, which may be explained by the much higher proportion of mothers aged <25 years in the cohort. Of the additional variables included in this study, perhaps the finding that DVA was associated with living in a sparsely populated area is the most interesting. Previous research links rurality with increased prevalence, severity, and duration of DVA. Possible reasons for this are that perpetrators may commit more chronic and severe DVA, mothers may have less access to social support networks in rural settings, there may be a lack of access to services such as hostels to potentially escape abusive behavior, and differential policing and health care may also have an impact [63,64]. The issue is complex, and there are evidently many plausible reasons to explain such an association. Additional data would be required to allow further investigation into these areas. Interestingly, deprivation, so often associated with risk of poor health, was not found to have a significant association with primary care–recorded DVA. Understanding this result would require further research but could indicate that using area-level measures of deprivation, which lack granularity, is not a suitable approach to examining the relationship between DVA and deprivation and that individual-level deprivation measures may be more robust for identifying risks associated with primary care–recorded DVA. Given underreporting of DVA within primary care settings, it may also be that those who actively engage with their GP and report DVA are not the most deprived.

At the time of writing, reliable ethnicity data were not available in the Cafcass Cymru data; therefore, this aspect was not explored in these analyses. Work is underway to improve the quality of ethnicity recording. However, recent research has highlighted the heterogeneity of the relationship between DVA and mental or physical health based on ethnicity [65], finding that non-Hispanic Black women and non-Hispanic White women who experienced DVA were more likely to experience poor mental health over time, whereas there was no association between DVA and mental health outcomes for Hispanic mothers. In addition, only Black mothers who experienced DVA were more likely to report more physical health problems over time.

### Study Strengths and Limitations

#### Strengths

To our knowledge, this is the first population-scale study using primary care records to identify DVA and examine risk factors for DVA among mothers in Wales. The study combined Cafcass Cymru data with health and demographic data to provide novel evidence on a group of mothers considered vulnerable and involved in family law court proceedings in Wales. Estimates of primary care–reported DVA prevalence are provided for a cohort of family court mothers as well as the matched cohort from the general population and validated against previous studies, which confirms underreporting of DVA in GP administrative data. Previously identified risk factors were recreated using linked administrative data based on previous research, thus facilitating national-scale analyses where research has previously been limited to smaller numbers.

#### Limitations

The study recreated NICE risk factors, which necessitated creation of proxy measures; for example, *long-term illness* was replaced in this study with *intellectual disability*, which prevents direct comparison with results reported for some risk factors. Such decisions were made based on pragmatism with regard to the availability of data and algorithms to recreate conditions. Future work could seek to develop a more robust version of the *long-term illness* measure as described by NICE.

As the study is cross-sectional, temporality cannot be determined; therefore, it is unclear whether the risk factors precede, and thus increase, the risk of DVA in GP records or vice versa. Longitudinal studies are recommended for future research, although such studies would also face underreporting issues, and it would be difficult to determine when DVA first occurred using GP administrative data. The 2-year prevalence of primary care–reported DVA of 5.73% (269/4695) reported in this study is likely a conservative figure. Estimating the prevalence of DVA is problematic owing to issues such as variation in screening and recording practices and definitions of DVA, as well as underreporting [66-70]. Underreporting of DVA is widely acknowledged owing to the numerous sensitive and complex reasons that can prevent those experiencing DVA from disclosing it to the police or in health care settings, some of which include fear that they will not be believed; fear of retaliation and further violence; economic dependence; fear of social services involvement, child protection proceedings, or loss of child custody; fear of a negative response from the police or health care professional; and lack of options to escape victimization [68,71-73]. The magnitude of underreporting is indicated by the CSEW, which estimated that only 18.4% of the women and 14.7% of the men who had experienced DVA between April 2017 and March 2018 reported it to the police [4]. Data linkage studies demonstrate substantial underrecording of DVA in GP data compared with CSEW data [9,12,13]. In addition, documentation of DVA by GP’s is inconsistent across the United Kingdom [69,70,74]. One early UK study on DVA in primary care found that DVA was documented in the medical records of only 17% of the women with self-reported DVA [11]. Barriers to recording DVA in primary care include attitudes of clinicians and those with exposure to DVA to recording and reporting DVA [11,67,69,74]; for example, GP’s have expressed concerns around preserving confidentiality and the time needed to inquire about DVA, and they have demonstrated a lack of awareness of national and local guidance on documenting DVA [67,69,74], whereas in 1 study, 20% of the women objected to screening for DVA [11].

### Implications for Research, Practice, and Policy

This research provides new evidence in terms of DVA prevalence and understanding of risk factors for a group of mothers traditionally hard to reach and considered vulnerable, some of whom may not be represented in official DVA statistics. The findings indicate that risk factors for DVA for mothers involved in public law family court proceedings differ from risk factors for DVA for the general population of women, as noted in the national NICE guidelines. With additional risk factors highlighted, there may be value in creating bespoke guidelines specifically relating to groups considered vulnerable, such as mothers involved in the family courts who were found to be much more likely to experience DVA than mothers in the general population.

The additional information may be useful in providing further information for those working with such individuals considered vulnerable in terms of policy setting or providing frontline assistance; for example, the increased risk of DVA for mothers living in sparsely populated areas could be used as evidence to improve support services in such areas. Increased risks associated with ED assault may be an obvious link but could be used to emphasize or provide additional services or signposting within EDs.

The findings corroborate underreporting of primary care–recorded DVA, an obvious limiting factor to this research. Purposefully improving such data collection would allow more robust analyses to corroborate or improve the evidence base to provide to stakeholders to allow more informed changes to be made to help those in need. Programs aimed at improved identification and reporting of DVA have been developed and run within GP practices [75], and research projects have reviewed and recommended improvements to DVA recording across health settings [70]. Programs such as these will improve the quality of data collection and subsequent use of the data for research.

### Conclusions

This study provides information on risk factors associated with DVA for a cohort of mothers involved in public family law proceedings who self-report to a GP compared with a general population comparison group and highlights the increased risk for this cohort of mothers considered vulnerable. Further evidence of the association between having a mental health condition and experiencing DVA should help to corroborate the necessity for continued and further resources targeted at specialist mental health and DVA support services. The additional evidence that this study adds in highlighting risk in sparsely populated areas should further inform service design.

An initial model including the general population of Wales shows common risk factors (NICE) can be reproduced with these data and analytical approach and highlights the increased association of primary care–recorded DVA for mothers involved with the family courts. Analysis of the same risk factors solely within the cohort of family court mothers emphasized key differences in associations with DVA. A final model that included additional risk factors helped to further explain some of the variability for the cohort, which adds more information and understanding of this population.

The results support the case for the use of administrative data to improve understanding of DVA risk factors, especially for groups considered vulnerable, to improve the evidence base and inform policy guidelines. Such groups that are hard to reach and considered vulnerable are more likely to be excluded from more traditionally gathered statistics, whereas, in this situation, their interaction with health services is recorded as standard; the inclusion of such individuals lessens implications of external validity and generalizability [76].

However, caution is urged in the interpretation of the results because the variability in individual situations and the complexity of lived experiences of DVA cannot be fully explained using the available data and modeling approaches used in this study. Improved reporting of DVA in GP administrative data would improve validity in future work. Nonetheless, accepting such limitations, the information provided is valuable and highlights risk factors that may be useful for policy makers and those services working with such mothers considered vulnerable.

## Appendix

Multimedia Appendix 1 Domestic violence and abuse Read code list.

## Abbreviations

ADHD: attention-deficit/hyperactivity disorder
ALF: anonymized linkage field
AOR: adjusted odds ratio
AUC: area under the receiver operating characteristic curve
CSEW: Crime Survey for England and Wales
DVA: domestic violence and abuse
ED: emergency department
EDDS: Emergency Department Dataset
GDPR: General Data Protection Regulation
GP: general practitioner
ICD-10: International Classification of Diseases, 10th Revision
LSOA: lower layer super output area
NICE: National Institute for Health and Care Excellence
ONS: Office for National Statistics
OR: odds ratio
PEDW: Patient Episode Database for Wales
RALF: residential anonymized linkage field
s.31: section 31 of the Children Act (1989)
SAIL: Secure Anonymised Information Linkage
WDSD: Welsh Demographic Service Dataset
WIMD: Welsh Index of Multiple Deprivation

## Data Availability

This study makes use of anonymized data held within the Secure Anonymised Information Linkage Databank. The authors would like to acknowledge all data providers who make anonymized data available for research. Data access requests, including Cafcass Cymru data, can be made via independent governance review panel applications [42].
