# Supplementary material for: Risk Factors Associated With Primary Care–Reported Domestic Violence for Women Involved in Family Law Care Proceedings: Data Linkage Observational Study
Source: J Med Internet Res. 2023 May 24;25:e42375. doi: 10.2196/42375 (PMC10248775; doi:10.2196/42375)
Supplement: Multimedia Appendix 1 [file jmir_v25i1e42375_app1.docx]

Appendix 1: DVA Read code list

Obtained from Jackson et al.[12].

Codes were truncated to the first five characters for use within SAIL.

| **Read code** | **Definition** |
| --- | --- |
| 14XG.00 | Victim of domestic abuse |
| Z415.00 | Domestic abuse counselling |
| 14XD.00 | History of domestic abuse |
| 67Ia.11 | Advice about domestic abuse |
| 14X3.00 | History of domestic violence |
| 8Hl7.00 | Referral to domestic violence advocate |
| 9GA..00 | Notification received of alleged domestic violence in household |
| 67Ia.00 | Advice about domestic violence |
| 14X8.00 | Victim of domestic violence |
| 14XE.00 | History of being victim of domestic violence |
| U3N0.00 | [X]Other maltreatment syndromes, by spouse or partner |
| U3M0.00 | [X]Neglect and abandonment, by spouse or partner |
| U3P0.00 | [X]Maltreatment, by spouse or partner |
| 9NDJ.00 | Police domestic incident report received |
| SN56300 | Battered wife |
| SN56100 | Battered woman, unspecified |
| 13HA.00 | Battered wife - history |
| 13Hm.00 | Subject of multi-agency risk assessment conference |
| 8T0b.00 | Referral to multi-agency risk assessment conference |
| 13HD.00 | Violent spouse |
| 67Ia.00 | Advice about domestic violence |
